# Ethereum blockchain for electronic health records: securing and streamlining patient management

**DOI:** 10.3389/fmed.2024.1434474

**Published:** 2024-09-25

**Authors:** J. S. Simi Mole, R. S. Shaji

**Affiliations:** ^1^St. Xavier’s Catholic College of Engineering, Nagercoil, India; ^2^All India Council for Technical Education, New Delhi, India

**Keywords:** EHR decentralized healthcare, Ethereum, scalability, interoperability, data privacy

## Abstract

Electronic health records (EHRs) are increasingly replacing traditional paper-based medical records due to their speed, security, and ability to eliminate redundant data. However, challenges such as EHR interoperability and privacy concerns remain unresolved. Blockchain, a distributed ledger technology comprising connected, encrypted data blocks, presents a promising solution. This study explores how blockchain technology can revolutionize hospital EHR management. Our proposed solution securely transfers medical records between patients and doctors using the InterPlanetary File System (IPFS) and the Ethereum platform. Utilizing smart contracts automates data transfers, ensuring patient anonymity and reducing computational complexity while securely storing patient data on the network. Patient records are stored locally on the Ganache server, with the front end managed using HTML, CSS, ReactJS, and JavaScript, and the backend developed in Solidity. Blockchain technologies combined with Role- Based access control instead of attribute -based access control. The system’s throughput increases linearly with the number of users and requests, enhancing the framework’s efficiency and scalability. The minimum recorded latency is 14 ms.

## 1 Introduction

Imagine having your entire medical history, from childhood vaccinations to current medications, stored securely in one place. Electronic Health Records (EHRs) make this a reality, revolutionizing the way healthcare providers manage patient information. EHRs are digital versions of traditional paper charts containing comprehensive and up-to-date patient data, including medical history, medications, allergies, test results, and treatment plans. EHRs enable seamless communication among healthcare providers, improve patient care, and enhance the overall healthcare experience. With EHRs, clinicians can access accurate and timely patient information, make informed decisions, reduce medical errors, and enhance patient engagement. EHRs are a crucial component of modern healthcare, transforming the way we manage and deliver patient care. As technology continues to evolve, EHRs will play an increasingly vital role in shaping the future of healthcare.

EHRs usually offer several positive implications for the healthcare sector. They can improve patient care by providing accurate and timely access to medical history, allergies, and medications. EHRs also increase efficiency by streamlining clinical workflows, reducing administrative burdens, and allowing clinicians to focus more on patient care. Additionally, they enhance decision-making through real-time access to patient data, which can lead to improved health outcomes. EHRs also foster enhanced patient engagement by empowering patients to take an active role in their care through secure messaging, appointment scheduling, and access to their medical records. Moreover, EHRs facilitate research, quality improvement, and public health surveillance by providing large-scale, de-identified data. Here are some potential impacts of EHRs:

1. Improved patient outcomes: EHRs can lead to better health outcomes by providing accurate and timely access to medical history, allergies, and medications.
2. Enhanced patient safety: EHRs can reduce medical errors, improve medication management, and enhance patient safety.
3. Increased efficiency: EHRs can streamline clinical workflows, reducing administrative burdens and allowing clinicians to focus on patient care.
4. Better decision-making: EHRs can provide real-time access to patient data, enabling informed decision-making and improved health outcomes.
5. Cost savings: EHRs can help reduce healthcare costs by minimizing unnecessary tests, improving resource allocation, and enhancing care coordination.

Before the advancement of modern technology, the healthcare industry used paper or card-based systems to keep patient health records. These systems lacked organization, security, efficiency, and tamper resistance. Redundant and replicated records across institutions made resolving these issues challenging. Between 2008 and 2018, approximately 200 million medical records were stolen and made public (1, 2). As healthcare systems become more digital to improve data management and access, patient data privacy concerns have also emerged (3, 4). Traditional EHR systems face problems with healthcare providers controlling and retaining patient health data, causing delays in data transmission and the timely provision of treatment (5). Another issue is the compatibility between various EHR systems. Due to these challenges, there is a need for an EHR system that enhances security and decentralizes patient data management. Studies have shown that providing patients with Internet access to their e-health data can increase satisfaction and convenience (6).

However, there are negative implications as well. EHRs are vulnerable to cyber threats, posing risks of data breaches that could compromise sensitive patient information. The high implementation costs of EHR systems can be a significant burden on healthcare organizations. EHRs can also contribute to clinician burnout due to cumbersome interfaces, excessive documentation requirements, and decreased face-to-face time with patients. Furthermore, reliance on EHR technology may lead to decreased critical thinking skills and clinical expertise. Lastly, if not designed and implemented with equity in mind, EHRs may exacerbate existing healthcare disparities. Even though the idea behind using EHR systems in hospitals or other healthcare settings was to raise the standard of treatment, these systems have several issues (research gap), as follows:

### 1.1 Interoperability

It is the method by which various information systems communicate with one another. The data needs to be interchangeable and functional for future uses. Health Information Exchange (HIE) or general data sharing is a significant feature of EHR systems.

Since different EHR systems are being implemented in different hospitals, their technical, functional, and terminological capabilities vary, making it impossible to create a single, globally recognized standard. Additionally, the medical records that are being shared should be technically interpreted, and the information that is understood may be used in other ways.

### 1.2 Information asymmetry

Information asymmetry, or one party having better access to information than the other, is regarded as the biggest issue in the healthcare industry today. This issue affects the healthcare industry in general and EHR systems in particular because hospitals and physicians have central access to patient records. A patient must go through a drawn-out and time-consuming procedure in order to access his medical records. The information is centralized within a single organization, and only hospitals or other healthcare organizations have access to these data.

### 1.3 Data breaches

Data breaches in the healthcare sector highlight the need for a more secure platform. Studies analyzing data breaches in EHR systems revealed that since October 2009, 173 million data entries have been compromised. Additionally, many EHR systems are not designed to meet patients’ needs and often face issues related to inefficiency and poor adaptation. The literature also indicates that the use of EHRs has introduced negative consequences for information processing. These challenges underscore the necessity of finding a platform that can transform the healthcare sector to be more patient-centered, such as blockchain.

Blockchain is a transparent and safe platform that guarantees the accuracy of patient data records. The structure for a decentralized platform that keeps patient medical records and allows authorized parties, including the patient, to access them is proposed in this study. We suggest utilizing an off-chain scaling technique to solve the scalability problem with blockchain, as it is not meant to hold massive amounts of data. This method solves the scalability issue by handling data storage through an underlying medium. Our suggested effort is to address the information asymmetry and data breach issues that EHR systems are now facing.

Thus, in this study, we offer a solution that can meet an EHR system’s security, privacy, interoperability, and performance requirements. The proposed technology allows patient data to be shared from any location at any time, provided the patient gives permission. We have considered all of the aforementioned requirements in this attempt to guarantee patient data confidentiality and privacy, achieve interoperability, and satisfy performance goals. By leveraging it’s sophisticated and dependable cryptography system, which permits data sharing between healthcare providers and grants patients control over their data, this work employs blockchain technology to meet these needs.

The proposed system aims to create an EHR application based on blockchain technology. Blockchain is a decentralized peer-to-peer network that facilitates secure data sharing. It offers benefits such as immutability, security, audibility, and transparency.

There are now several blockchain platforms, including Cardano, Ethereum, QTUM, and NEO. The Ethereum platform is a frontrunner in the implementation of smart contracts and blockchain-based applications. It is widely acknowledged as an advanced blockchain platforms that are capable of carrying out a variety of functions (such as security data exchange) that could be useful to a wide range of industries, not just the financial sector. Consequently, we incorporate this platform into our suggested blockchain-based architecture. Using this platform, the works proposed a patient-centric framework that stores patient data in blockchain smart contracts and operates in a decentralized manner.

Transaction details with security and privacy features are communicated through the smart contract after it is launched. Moreover, the proposed modifications to the transactions can be mined and distributed to all of the decentralized systems.

Thus, the primary goals of the suggested efforts are:

1. Creating and implementing a frontend platform for an EHR online portal with a patient-centric focus.
2. Connecting the patient-focused EHR mentioned above to the Ethereum blockchain and its Smart Contract.
3. Ensuring the privacy, security, consistency, and accessibility of a patient’s health record, as determined by the patient, across healthcare providers.
4. Test the suggested blockchain-enabled framework’s interoperability, security, and privacy.

Healthcare professionals can look for patient data using our suggested framework and ask for permission to access it. Patients control their data, allowing for faster data transfer between EHR systems. Data about every patient is kept on the peer-to-peer node ledger. Ganache is used to test the suggested framework on a private Ethereum network. The outcomes demonstrate how well the system performs in terms of security, privacy, and interoperability.

Section 2 provides an overview of related work. Section 3 outlines the preliminaries used for the proposed framework. Section 4 details the system architecture and its implementation. Section 5 presents the performance assessment of the proposed system and its results. Section 6 discusses the findings and addresses ethical considerations. Finally, Section 7 concludes the study and suggests directions for future work.

## 2 Related work

When Nakamoto created blockchain technology, the main goal was to create decentralized, cryptographically secure money that would be useful for financial transactions. In the end, the blockchain idea was applied in many other domains. The healthcare industry is one of them, and they plan to employ it. A study by Gordon and Catalini concentrated on the ways that blockchain technology will help the healthcare industry. The study also discussed the difficulties or impediments to the use of blockchain technology, including the massive volume of medical records, privacy and security concerns, and patient involvement (7).

Rahmadika and Rhee (8) suggest a theoretical model that relies on blockchain knowledge in a peer-to-peer system to manage the private medical data collected from several healthcare providers. In addition to facilitating the effective sharing of personal healthcare information between patients and healthcare providers, it ensures data security and integrity. Immaculate data records are provided by the blockchain without the need for an intermediary.

Kim and Lee (9) discussed a situation in which a third party wants access to the questionnaire data; in this case, the patient’s consent must be obtained, as demanded by the doctor, for the third person to view the data. In this study, a blockchain-based medical data storage system was suggested. The system can protect patient privacy in addition to ensuring the authenticity and verifiability of medical data.

Medical records that are kept on the cloud in private and are only available to the patient are covered in Samarin (10). It is clear from this that patients now fully own their medical records. It ignores, nevertheless, the necessity of disclosing medical information to several parties, including healthcare providers. Additionally, when a record is transferred to another party, this work uses a deposit box to alleviate the interoperability issue. Nevertheless, the idea put forward in this work does not deal with the circumstance where a physician must withhold a patient’s medical records from everyone, including the patient. Furthermore, the security component of medical records is not included in this study.

To facilitate the reuse and interchange of various patient records within and across units of the same organization, as well as between them, the service-oriented architecture (SOA) was proposed in Li (11). To address interoperability, reusability, and security issues with PHR systems, this method was also utilized for the integration of EHR and Personal Health Record (PHR) systems. The compatibility with other PHR systems and the incomplete attention to patient data security and privacy provide a barrier to this method.

Blockchain is utilized in Azaria et al. (12) to store medical records. Patients can securely access their medical records with the suggested solution. Patients are fully informed of any changes made to their records thanks to the permission management tool, which additionally verifies the type of data that should be given to each blockchain miner. This architecture employed proof-of-work (POW) consensus techniques to verify newly produced blocks in the blockchain and smart contracts. The system can receive data from several sources. This system’s failure stemmed from its failure to address database security concerns.

Using a Hadoop database, Sahoo and Baruah (13) presented a scalable blockchain platform. They suggested combining the decentralization offered by blockchain technology with the scalability of the underlying Hadoop database to address the scalability issue of blockchain. They employed the technique to store blocks in the Hadoop database; the blockchain sits atop this framework and has all the necessary dependencies, but the Hadoop database stores the blocks, which increases the scalability of the blockchain technology. This study suggests using the Hadoop database system in conjunction with SHA3-256 hashing for transactions and blocks to address the scalability issue of the blockchain platform. Java was the programming language utilized to create this architecture. This study helped to clarify how blockchain can be utilized in conjunction with other scalable platforms to enhance or address the scalability issues on this particular platform.

Velmurugan and Prakash (14) address that though Hyperledger blockchain technology is decentralized and uses encryption, it offers high security, but it also has scalability issues. As the quantity of health records increases, the blockchain may become increasingly overburdened, which could impact the efficiency and speed of data transfers. A blockchain system that acts as a middleman between users and the collection of private information shared by everybody was suggested in Xia et al. (15). There has been a comparison between this technology and other blockchain platforms like Bitcoin. This system uses encryption techniques to validate patient data. The system’s end-to-end test demonstrated the scalability, effectiveness, and lightweight nature of the employed technique. It is also mentioned that more research on authentication and communication protocols is required in the future. Privacy and security assessments were additionally intended to be carried out, particularly concerning external access to private blockchain networks (16).

The Ethereum blockchain platform is utilized in Shahnaz et al. (17) and Zhou et al. (18), as Table 1 illustrates. The suggested technology is referred to as MIStore Zhou et al. (18), and it is a blockchain-based medical insurance storage system. This uses less CPU and memory. The Ethereum blockchain is utilized in Shahnaz et al. (17) for electronic medical and health records. Both consider compatibility, security, privacy, interoperability, and performance based on the Ethereum blockchain and develop a framework that can meet all the requirements. The work presented in this study moves towards the idea that patients will only own their own data and grant permission for it to be processed by a third party.

**Table 1:** A summary of the pertinent research utilizing the suggested Ethereum-based blockchain.

Citation	Platform used	Features present and features not present ×			
		Interoperability	Response time	Security	Compatibility
Azaria et al. (12)	MedRec blockchain	✓	✓	×	✓
Sahoo and Baruah, (13)	Permissioned blockchain with cloud	✓	✓	×	✓
Velmurugan et al. (14)	Ethereum blockchain	✓	✓	✓	✓
Roehrs et al. (16)	OmniPHR Architecture model	✓	✓	✓	✓
Shahnaz et al. (17)	Ethereum blockchain	✓	✓	✓	✓
Zhou et al. (18)	Ethereum blockchain	✓	✓	×	×
Mandarino et al. (19)	Ethereum blockchain	✓	✓	✓	✓
Komala and Arun Kumar, (20)	Ethereum blockchain	✓	✓	✓	✓

As per Valerio and Giuseppe (19), while decentralization, transparency, security, and immutability are some of the attractive qualities that blockchain technology offers, it also confronts many serious obstacles, such as those on scalability, privacy, and interoperability, all of which need to be carefully considered and resolved. The degree to which these constraints are addressed could have a significant impact on the acceptability and success of blockchain-based applications. Hence, they suggested a result that allows the adoption of blockchain for sharing EHRs, with special attention to the option of using public blockchains such as Ethereum.

Komala and Arun Kumar (20) suggested an Ethereum-based system, and its salient features encompass decentralized data storage, encryption to ensure data privacy, access control measures, and unchangeable audit trails. Patients can maintain ownership and control over their medical records by implementing smart contracts, giving or removing access to healthcare professionals as needed. Furthermore, the system makes it easier for authorized parties to share data securely and seamlessly, which enhances interoperability while preserving the security and integrity of the data. A summary of the pertinent research utilizing the suggested Ethereum-based blockchain is given in Table 1.

To ensure the smooth operation of the proposed framework, certain constraints must be taken into account. Table 2 outlines some of the challenges that the new system needs to address compared to the existing one.

**Table 2:** The challenges that the suggested system must take into account.

References	Constraints that need to be resolved in the proposed system
Roehrs et al. (16)	Scalability, data secrecy, and safety
Dwivedi et al. (34)	Data confidentiality and safety, interoperability, and data scalability
Shen et al. (35)	Data sharing, data integrity, scalability, and data secrecy
Jamil et al. (36)	Global data access and interoperability
Margheri et al. (37)	Data secrecy and safety
Zhuang et al. (38)	Data secrecy, safety, data leak, and scalability
Alzahrani et al. (39)	Scalability
Silva et al. (40)	Lack of access control
Gunturu et al. (41)	Lack of technical skills
Dubovitskaya et al. (42)	Lack of data storage facilities

## 3 Preliminaries used for the suggested framework

It provides an explanation of the software platforms that were used to build this framework while accounting for the challenges listed above. IPFS and Ethereum are crucial platforms for putting this idea into practice.

### 3.1 Ethereum

Ethereum is a distributed blockchain network that leverages the blockchain technology initially introduced by the cryptocurrency Bitcoin. Officially released in 2015, Ethereum was designed to create an open-source, programmable blockchain system for trustless smart contracts. It also incorporates peer-to-peer connectivity, enabling decentralized distribution (21). The native cryptocurrency of the Ethereum network is Ether, which can be transferred between accounts on the blockchain (22). Additionally, Ethereum provides developers with solidity, a programming language specifically designed for creating and executing smart contracts on the blockchain.

### 3.2 Data contract

Ethereum contracts describe how an outside party can communicate with Ethereum. An external user may utilize it to edit the information or record that is kept on the Ethereum blockchain network. The following components are present in an Ethereum transaction (23).

- To: the recipient of the communication, who likewise has a 20-byte address.
- From: The sender of the message.
- Value: The amount of money sent from one party (sender) to another party (receiver).
- Information: Holds the text delivered to the receiver.
- Gas: The sender must pay a fee on the Ethereum blockchain called Gas in order to complete a transaction. Every transaction includes the gas price and limit.
- Gas price: The amount of money the transaction ender is prepared to spend on gas.
- Gas limit: Most of the gas was allowed to be used in this transaction.

### 3.3 Smart contract

A smart contract is a segment of code that can be deployed on the blockchain to perform various operations. This part of the program is executed when users initiate transactions or send communications (43). Smart contracts are resistant to tampering and modifications since they operate directly on the blockchain. The Solidity language is widely used in smart contract programming, enabling developers to script any desired blockchain functionality. Once the necessary actions are defined, the program is compiled into Ethereum Virtual Machine (EVM) bytecode. This bytecode can then be executed and implemented on the Ethereum blockchain. Solidity, the language provided by Ethereum for writing smart contracts, integrates features from Python and JavaScript, making it accessible to developers familiar with these languages.

### 3.4 Ethereum virtual machine

The apps that are generated by smart contracts are run on the EVM.

### 3.5 Interplanetery file system

IPFS is a protocol that stores data on a peer-to-peer network because IPFS provides safe data storage and prevents data manipulation. To prevent data alteration, it uses a cryptographic identity; any attempt to alter the data on IPFS necessitates altering the identifier. Each data file that is kept on IPFS has a cryptographically generated hash value. It serves as a unique identifier for IPFS data storage (24).

### 3.6 MetaMask

With the help of the well-known browser extension MetaMask, users may communicate with decentralized Ethereum apps right from their browsers. By offering an intuitive user interface for managing Ethereum accounts, communicating with smart contracts, and completing transactions, it operates as a bridge between users and the Ethereum network.

Some healthcare organizations utilize the Fast Healthcare Interoperability Resources (FHIR) to address the problem of interoperability in EHRs, while others use the HL7 2.x standard or the Clinical Document Architecture (CDA) standard for data interchange. Interoperability is harmed directly by these disparate data standards. In this work, we leverage blockchain technology to overcome this obstacle by using APIs to access data. By doing this, data formats are standardized and may now be transmitted in a single format regardless of an EHR’s capabilities.

## 4 System architecture and implementation

### 4.1 System architecture

We suggested utilizing the React web framework to create an EHR system on a web application on the Ethereum blockchain. The program will be connected with a blockchain-based Ethereum to secure patient data through dependable transactions using IPFS and Metamask. Healthcare practitioners will find this model very helpful in effectively maintaining patient records. Smart contracts, which are self-executing programs designed to automate necessary processes in an application, will be employed to regulate the transfer procedure. The terms and circumstances of the transfer, as well as the parties (patient, doctor) and the data to be shared, will be outlined in the smart contract. It will guarantee patient data privacy and security throughout the transfer procedure. The blockchain will be used to decentralize all data. It entails setting up a blockchain network, utilizing smart contracts to automate data transfers between patients and doctors, and securely storing patient data on the network. To guarantee that data are only accessible by the person who created the account on this system, smart contracts regulate access and data transfers. The blockchain network will store this information, enabling the creation of an unchangeable and transparent record of the transfer. Ganache assigns a unique ID to every patient. The doctor can access and search the patient’s medical records in the EHR system with that ID.

The admin can only add doctors and patient data to the system using the Ganache ID, and in the admin dashboard, the admin can view the doctors and patient data. To add the patient details, the patient needs to register in the system using the Ethereum ID, which is done by the organization’s admin. On the doctor’s page, they can consult and view appointments and patient records and prescribe medicine to the patient. Using the patient ID, the records can be accessed by the doctor and the patient. The user’s (patient or doctor) data is encrypted and saved as Ethereum blocks when it is stored in the blockchain. They use a two-way authentication procedure, such as obtaining a secret key produced by Metamask, to save data for the records to be safely kept under the blockchain. Systems for EHRs are exclusive and are intended to be decentralized. The entire process may be made visible and verifiable from beginning to end by storing the records on a blockchain. Thus, the patient’s medical records are easily transferred between the patient and the doctor.

**Figure 1:**
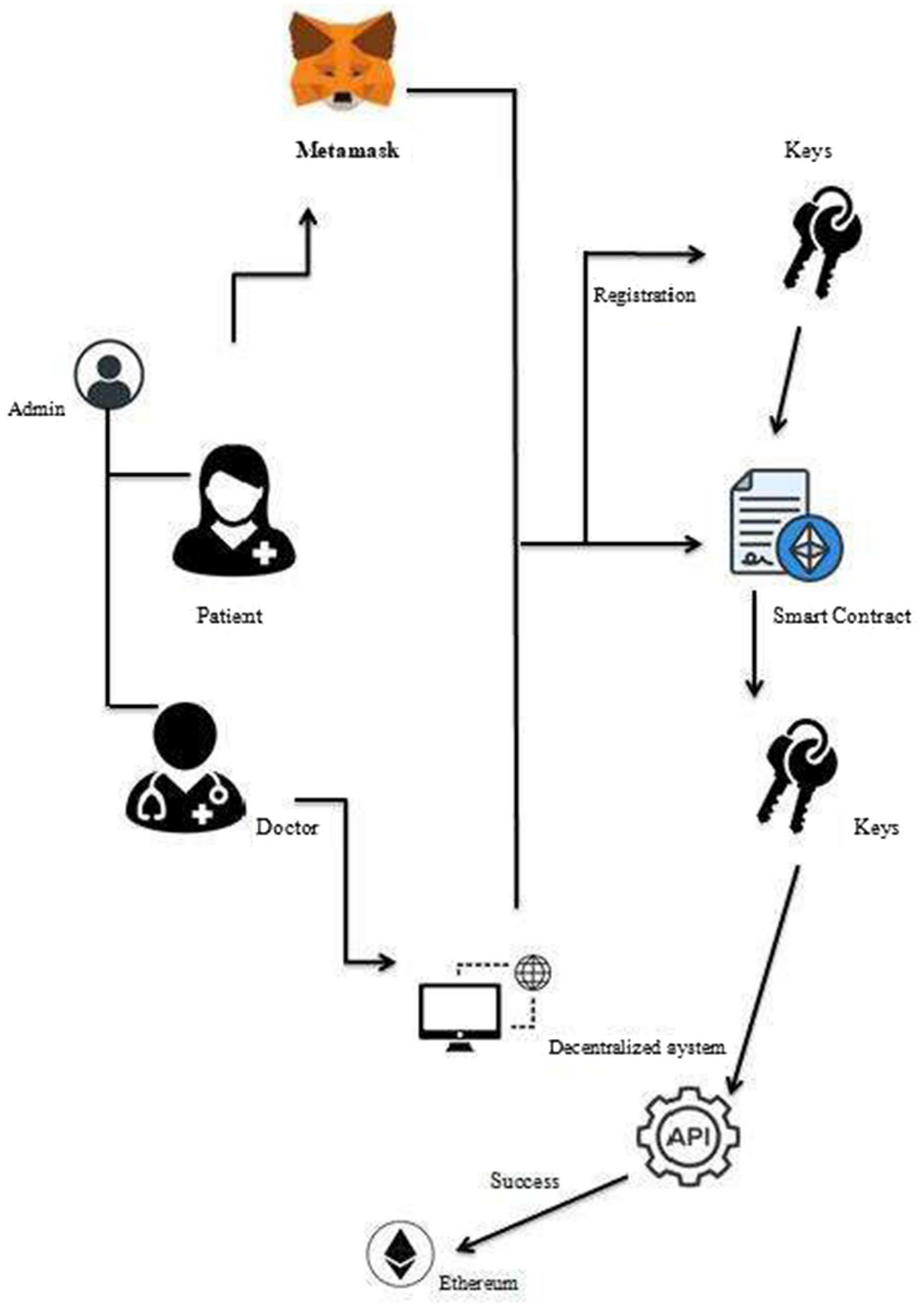
Ethereum blockchain-based EHR management system.

Figure 1 depicts the system structure when a patient chooses to examine their medical records through MetaMask, or the healthcare system’s decentralized website. By retrieving the private key from the Ethereum wallet, the user is automatically logged in. In this system, Ethereum wallets act as cold storage, minimizing the risk of compromise compared to hot wallets. Furthermore, if the device is missing, users can simply receive a replacement without being penalized for losing their medical records. The wallet can be used in the same way to sign any document or for verification purposes. This wallet can be used for multi-party patient verification. It can be used to create both a role-based access control system for records and a blockchain-based distributed property identification system. In the event of a medical emergency, a similar multiple-party permission process can be used to obtain access to the patient’s records.

The proposed system works in three layers: the user layer, the blockchain layer, and the system execution layer.

#### 4.1.1 User layer

The users of this system could be patients, doctors, and administrative staff. The main task of these users would be to interact with the system and perform basic tasks such as creating, reading, updating, and deleting medical records. The users using this system would be accessing the system’s functionality through a browser, which, in technical terms, we refer to as DApp browser.

#### 4.1.2 Blockchain layer

The blockchain layer is the next in the system. It contains the code or transactions that allow users to communicate with DApps, which function on the blockchain. The system includes the following transactions:

**Add records** generates a patient’s medical records in the DApp. It includes the fields ID, name, blood group, and IPFS hash. The patient’s basic medical records are saved with the IPFS hash of the uploaded file, which may include test results or other medical records.

**Update records** updates the patient’s medical records. This simply changes the patient’s basic information, not the IPFS hash. To preserve record security, the IPFS hash cannot be updated.

**Examine records** allow the user to examine a patient’s medical records saved in DApp, and this function is used by both doctors and patients. The patient can examine his records after the system authenticates that he is only viewing his own medical records. For this aim, the system leverages the patient’s public account address to ensure that only relevant medical records are shown to the patient.

**Delete records** allow the user to delete a patient’s record. The users, in this case, are doctors who can remove any patient information stored on the blockchain.

**Grant provides access** to each of the above-mentioned transactions; for example, only the doctor or nursing staff can alter or add to the patient’s records. As a result, only these people have access to add and alter records. Furthermore, the patient will be able to view his medical records but not add or change them.

#### 4.1.3 System execution layer

The system was implemented utilizing Ethereum and its dependencies. This layer includes the smart contracts. Smart contracts are an important part of DApps, and they are used to perform the basic transactions specified above.

**Figure 2:**
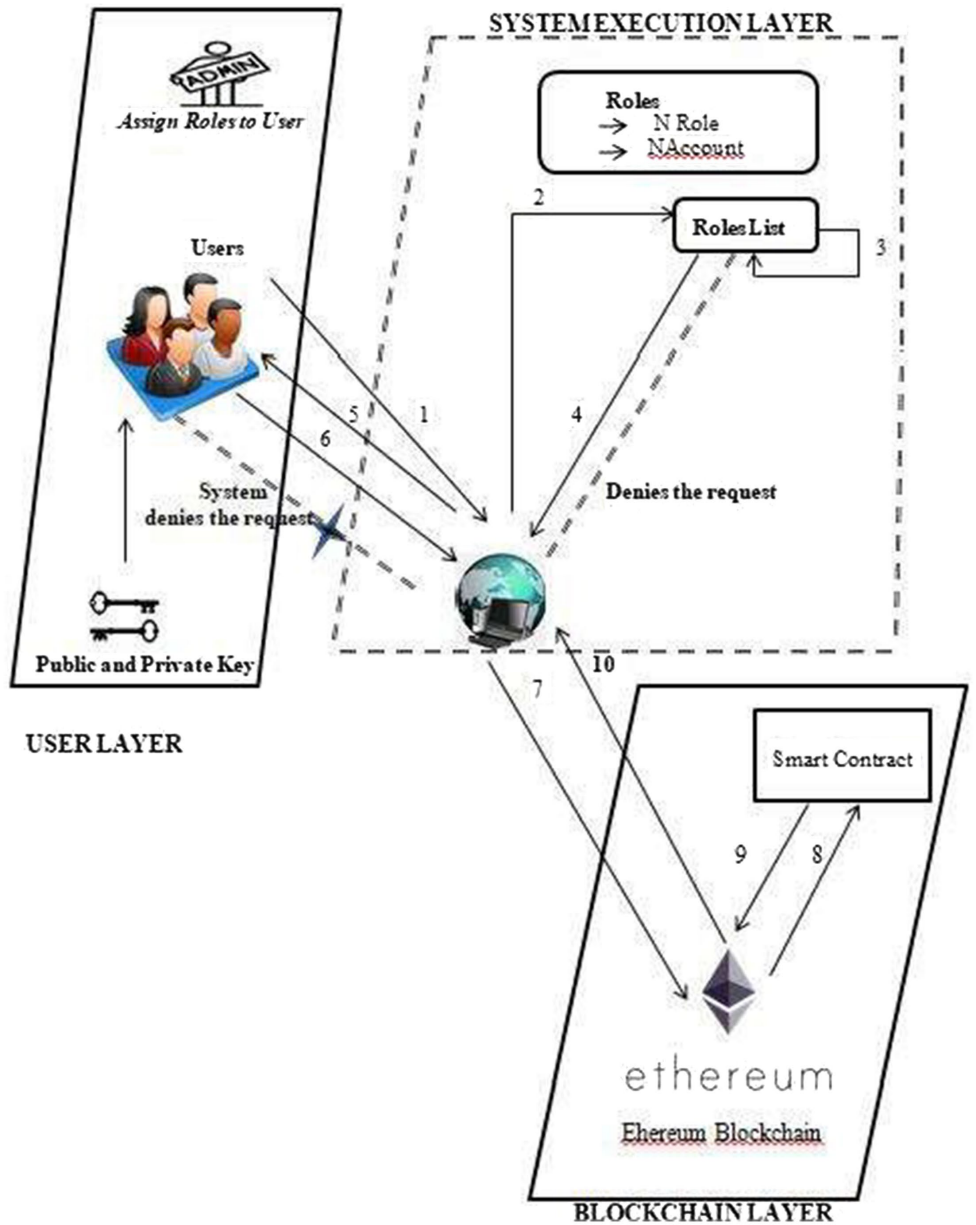
User-DApp interaction with system design.

Figure 2 depicts the basic usage scenario of the proposed framework, which operates across three layers. The system involves two primary entities: administrator and user. Users are further divided into two groups within the framework: doctors and patients. The system administrator, a member of the hospital’s administrative staff, is responsible for assigning roles to these users. Specifically, the administrator defines granular access permissions for the two main user groups—doctors and patients—ensuring appropriate access control within the system. Therefore, the first activity is for the administrator to assign roles, and this includes the role name (NRole) and AccountAddress (NAccount) of the user who is being assigned that role. Every user of this proposed system would have a role name and account address for using the system. As a result, the role name and account address that the administrator provides to this user are kept in a roles list for validation purposes and are used later. Following the role assignment, users will now carry out the following tasks shown in Figure 2:

1 Users request to execute certain actions on the proposed system when they want to.

2&3 Upon successful validation, the system verifies the user’s role name and account address from the RolesList and permits them to carry out those tasks.

4&5 Upon successful validation, the system verifies the user’s role name and address from the roles list and authorizes them to perform those actions.

6&7 The system would store the information on Ethereum after the jobs were finished.

8&9 A blockchain that would use such information to execute transactions.

10 Upon transaction confirmation, the blockchain layer notifies the system with a message of success that users can view on the DApp browser, which shows the whole framework proposal.

### 4.2 Implementation

The algorithm outlines how the patient interacts with and records functions within the Smart Contract. It includes five key functions: assigning roles, inputting data, retrieving data, modifying data, and deleting data. Each function plays a crucial role in managing the patient’s information securely and efficiently within the blockchain-based system.

#### 4.2.1 Allot roles

This means that it allows for the creation of new user roles and assigns them to specific accounts.

#### 4.2.2 Input data

The code attempts to add data to a patient’s record if the sender is a doctor. Otherwise, the session will be aborted. This means that only someone who has been identified as a doctor can access this function and add data to a patient’s record.

#### 4.2.3 Get data

The code is a function that allows a doctor or patient to view a specific patient’s record by providing the patient’s EID, and if the EID is valid, the function will retrieve and return the patient’s record to the requesting account. If the EID is not valid, the session will be aborted.

#### 4.2.4 Modify data

Overall, this function shows how programming concepts can be applied in real-world scenarios, such as managing medical records efficiently while maintaining security measures. The code attempts to modify the data of a patient’s record if the sender is a doctor and the provided patient’s EID and name match the record; otherwise, it will abort the session.

#### 4.2.5 Delete data

This function allows a doctor to delete a patient’s record if they are the sender and the patient’s EID matches. Otherwise, it will return a failed message or abort the session.

##### Algorithm 1 Smart contract to obtain patient records

**Table:** 

**1. Allot Roles:** **Function** define Roles (NRole, NAccount) add fresh role and account in the mapping of roles **end** function **2. Input Data:** **Function** Input Patient Data (var. 1,var2…) **if** (message. Sender = =doctor) **then** input data to a patient’s record **else** Terminate session **end if** **end function** **3. Get Data:** Function Get Patient Record (patient Eid) **if**(message.sender==doctor||patient)**then** **if** (patient Eid)==true **then** receive data from patient (Eid) return (patient record) **else** terminate session **end if** **end if** **end function** **4. Modify Data:** **function** Modify Patient Record (var1,var. 2…) **if** (message.sender==doctor) **then** **if**(id==patient Eid && name==patient name) then Update information to a specific patient’s record return true **else** return false **end if** **else** Abort session **end if** **end function** **5. Delete Data:** **function** Delete Patient Record (patient Eid) **if** (message.sender= =doctor) **then** **if** (Eid= =patient Eid) **then** delete a particular patient’s record return true **else** return false **end if** **else** Terminate session **end if** **end function**

### 4.3 Proposed framework working example

Let us examine the following example to see how each transaction happens in the Ethereum blockchain in terms of transaction time.

The transaction time taken for each transaction on the Ethereum blockchain depends on its current network traffic and its gas price. Normally, each block is included in the Ethereum blockchain only after a transaction is completed, and a new block is produced every 15 s. However, this becomes more varied when there is high network congestion. To speed up the transaction, users can increase the gas price, which, in turn, means high transaction fees. The duration of a smart contract transaction is 38 s, contingent upon the cost of gas that is specified throughout the transaction. Ethereum has a gas restriction rather than a block size limit.

As a result, depending on the volume of data supplied for the add function, an add function transaction in algorithm one will take 1 to 2 min. To obtain the data function or view the patient record. It will take approximately 45–50 s.

Let us examine the following example to better understand how the algorithm functions in terms of transaction size.

Let the average number of transactions per hour be 53,299


Blocksperhouronaverage=262

$$\begin{array}{l} \begin{array}{l} \mathrm{Average hourly transaction volume}/\\ \mathrm{the average quantity of blocks in an hour}=53,299/262 \end{array}\\ \;=203\;\mathrm{is the average number of transactions}\;\mathrm{per}\;\mathrm{block} \end{array}$$

$$\begin{array}{l} \begin{array}{l} \mathrm{Block size}/\mathrm{average number of transactions in}\;a\;\mathrm{block}\\ =55.90\;KB/203 \end{array}\\ \;=0.27\;KB\;\mathrm{is the average transaction size} \end{array}$$

Applying the above computations, an approximate value of 0.3 KB is found for the average transaction size. Additionally, bear in mind that the numbers above are exclusive to the Ethereum blockchain network and are current as of right now.

### 4.4 System configuration

The proposed system is designed using the following configuration for better performance.

- Processor: AMD PRO
- SSD: 512GB
- RAM: 4GB
- Input devices: Keyboard, Mouse
- Operating system: Windows 11
- Frontend: Angular, Bootstrap
- Backend: IPFS, Ganache, Truffle, Metamask
- Language used: Solidity, JavaScript

### 4.5 Performance evaluation

#### 4.5.1 Evaluation metrics

Total transaction time, latency, and output are the different metrics considered for evaluation for the suggested system, and they are as defined below:

##### 4.5.1.1 Total transaction time

It depends on transaction deployment time (Dx1) and transaction completion time (Cx2), i.e., the total time to complete a transaction (Cx2) and its deployment in the Ethereum blockchain (Dx1) (in seconds).

Max (Cx2)-Min (Dx1)

##### 4.5.1.2 Output

The volume of data moved from one location to another within a specified time frame.

##### 4.5.1.3 Latency

This refers to delays that occur when one element waits for another to respond to an action. It was referred to as the time gap between the transaction’s deployment and completion times.

#### 4.5.2 Transaction fee calculation [Transaction]

We can also calculate the price or charge related to the different system interactions.

Generally speaking, Ethereum calculates transaction fees in “ETH, with its units *wei and gwei*.

The method to compute Ethereum transaction fees is:


Transactionfee=GasConsumed×GasPrice

With the suggested gas consumption of 21,000 and the gas price of 21 Gwei, we can compute the transaction charge.


$$\begin{array}{l} \mathrm{Therefore},\mathrm{Transaction}\;\mathrm{Fee}=21,000\times 21\\ \;=441,000\;\mathrm{Gwei} \end{array}$$

Additionally, we would apply the following formula to determine the 1ether transaction fee:


1Ether=1,000,000,000Gwei

$$\begin{array}{l} \mathrm{One}-\mathrm{Ether Transaction}\;\mathrm{Fee}=441,000/1000,000,000\;\mathrm{Gwei}\\ \;=0.00041\;\mathrm{Gwei} \end{array}$$

In Ethereum, the transaction cost is simply the price of gas multiplied by the amount of gas consumed during the transaction (25). At least one of these values should be reduced to reduce transaction costs. In general, the price of gas should not be controlled by Ethereum smart contracts but should be chosen by the user when creating a transaction. This allows users to choose between low-cost transactions and quickly add their transactions to the blockchain. The amount of gas consumed, on the other hand, is a variable that smart contract developers can and should optimize. Ethereum’s transactional payment system is based on the idea that the use of computing, bandwidth, or storage resources costs gas. Thus, making the contract less resource-intensive in these matters also reduces gas consumption, which represents our goal.

The transaction fees for every function of the proposed system Algorithm 1 with 1 Gwei are shown in Table 3.

**Table 3:** The proposed system’s transaction fees.

Various transactions performed	Gas used	Size (BYTES)	Fee(ETH)
Allot roles	23,112	132	0.02311
Input data	29,768	548	0.02976
Get data	22,952	122	0.02295
Modify data	27,720	420	0.02772
Delete data	11,556	132	0.01155
Transaction Fee = Gas Used*Gas Price(1Gwei)			

As an alternative to deploying on the public Ethereum blockchain, the Plasma sub-blockchains proposed by Poon and Buterin (26) may provide a viable platform for future deployment. Plasma sub-chains can provide a similar execution environment that is linked to the main Ethereum chain but with reduced transaction requirements, leading to lower transaction costs. Another option would be to create a separate instance of the Ethereum blockchain. While this would allow transactions to be completed at a significantly lower cost than the canonical Ethereum chain, or potentially without transaction fees, the lack of support for Ethereum’s native cryptocurrency and the security provided by the canonical chain could present challenges.

Several approaches have been proposed to minimize Ethereum transaction fees. One solution is to optimize transaction fees by determining the minimum price a user must pay to process their transaction within a certain period of time (27). Another approach is to move most of the contract execution off-chain and trigger on-chain execution only if the parties disagree, reducing gas usage by 40.09% (28). In addition, the Optimistic Aggregation Technique (ORU) allows the delegation of computing from the main Ethereum blockchain to an untrusted remote system, reducing transaction fees up to 20 times (29). Additionally, an algorithm based on Max-Min Fairness was developed to distribute Ether in a manner that maximizes user fairness and minimizes transaction costs (30). These approaches aim to lower transaction fees and optimize the cost-effectiveness of transactions on the Ethereum blockchain.

## 5 Proposed system performance assessment and results

### 5.1 Performance assessment

We conducted a performance evaluation to determine how effectively our proposed framework would perform in an actual setting where multiple operators would be using it for various tasks. The evaluation parameters listed below serve as the foundation for performance assessments.

#### 5.1.1 The average time for execution

As the number of transactions increases, so does the execution time. These transactions are carried out for several purposes by the smart contract, the algorithm of which is also described. The time it takes to perform the Allot Roles, Input Patient Records, and Get Patient Records operations on a single-user system is 16 s, 1 min 50 s, and 45 s, respectively. When more users are utilizing the system at once, this time will grow.

#### 5.1.2 Throughput

**Figure 3:**
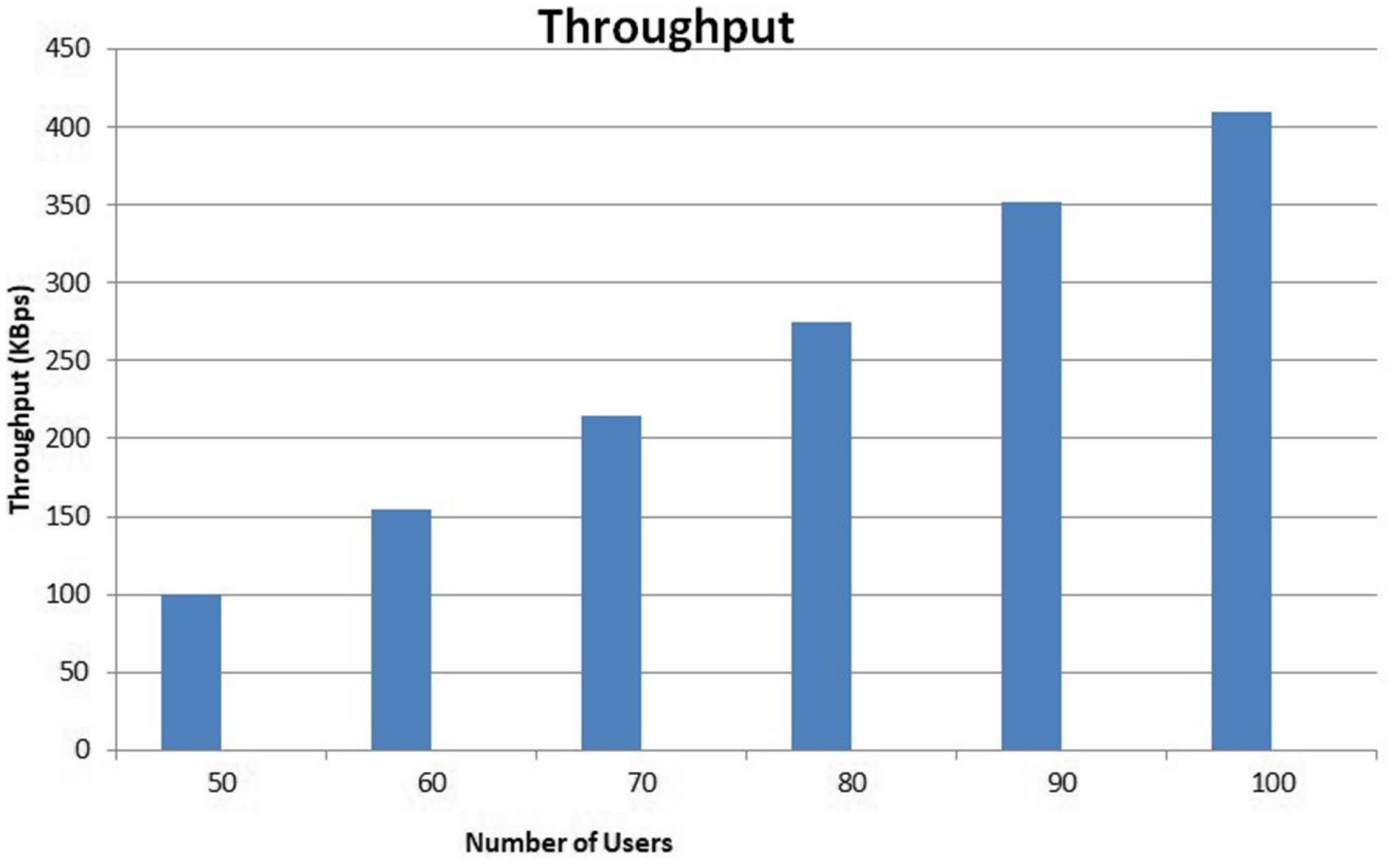
The proposed framework’s throughput.

Algorithm 1 shows several functions added in the Smart Contract for the planned system. Here, it simulates the number of users from 50 user’s to100users with a period of 5 to 20 s. The throughput evaluation used data/time, or KB/s, units to represent throughput. We evaluated the system’s performance while working with the above-specified user count, and throughput analysis was done at the conclusion. Figure 3 illustrates the throughput of the suggested structure.

During this experiment, it was discovered that the system’s throughput increased linearly with an increase in the number of users and requests. The linear development of the throughput indicates the effectiveness of the proposed framework.

#### 5.1.3 Average latency

**Figure 4:**
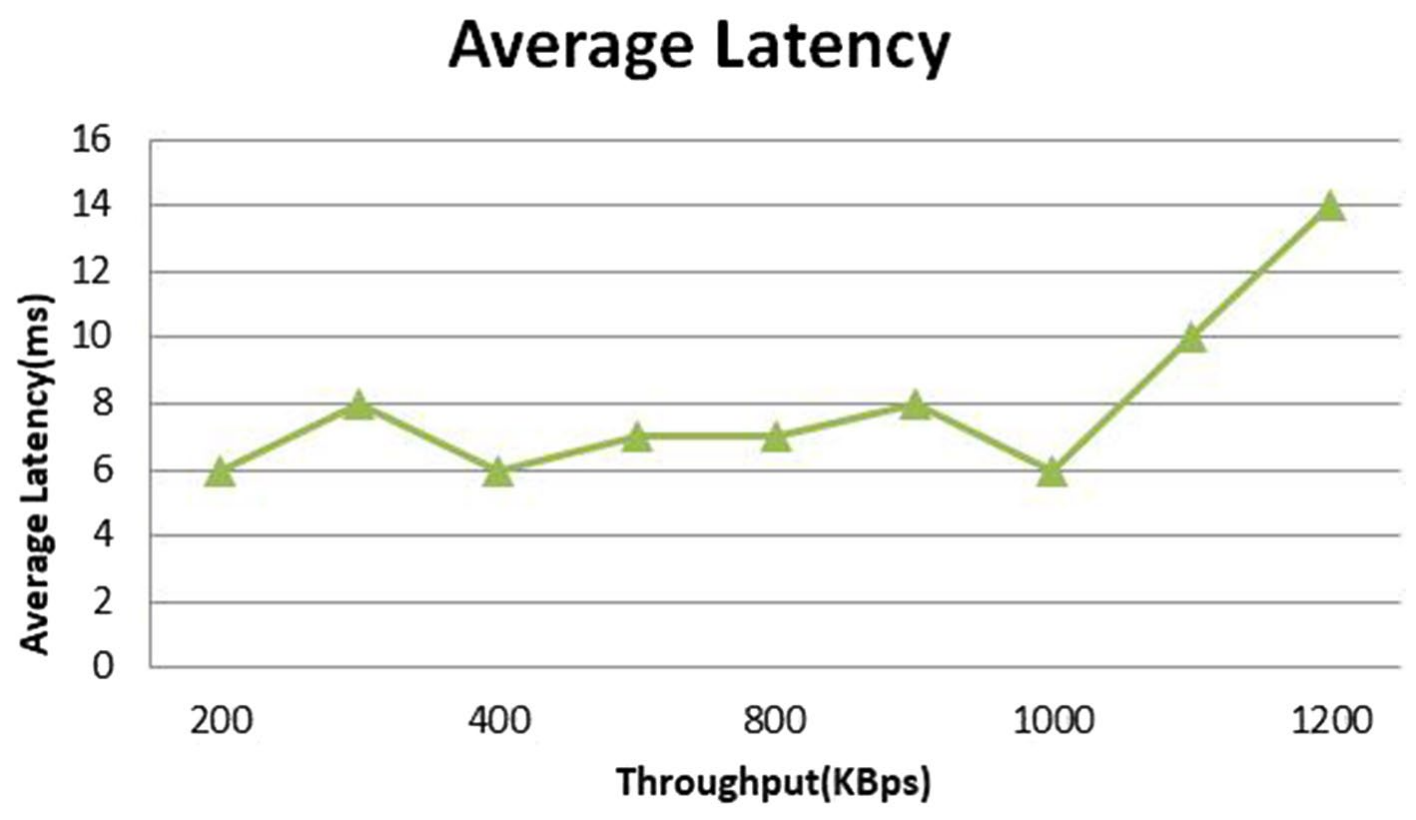
An overview of the typical latency.

As previously mentioned, latency was defined as the variance between the transaction’s deployment and completion times. Milliseconds are used to quantify latency. Figure 4 displays the system’s average latency overview and the suggested system’s throughput. The latency recorded here is 14 ms. these estimates suggest that our system is processing approximately 150–200 transactions per second (assuming an average transaction size of 100–150 bytes) with an average latency of 14 ms and may vary depending on the specific blockchain, network conditions, and system architecture.

Based on theoretical calculation, a transaction size of 150 bytes, a throughput of 1200KBps, and a latency of 14 ms are found to be compatible with each other. This suggests that the system can process a significant number of transactions (approximately 8,000 per second) with relatively small transaction size and low latency.

### 5.2 Security analysis

Using the STRIDE framework (represented by assets, threats, vulnerabilities, mitigations, and security controls), we carried out a rudimentary threat modeling effort to find potential security risks and weaknesses in our EHR system. The term “asset” refers to EHRs, private patient data, and private medical data. Threats include information regarding malware attacks (elevation of privilege), data breaches (repudiation and denial of service), and unauthorized access to EHRs (spoofing and tampering). Vulnerabilities describe outdated software components, invalidated user input, and weak access controls. Mitigations stipulate implementing robust access controls, validating user input and sanitized data, and ensuring up-to-date software components and regular security patches. Details about the encryption used for data in transit and at rest, regular penetration tests and security audits, and an incident response strategy are all part of the security controls.

### 5.3 Results

The output of the suggested work is shown below.

**Figure 5:**
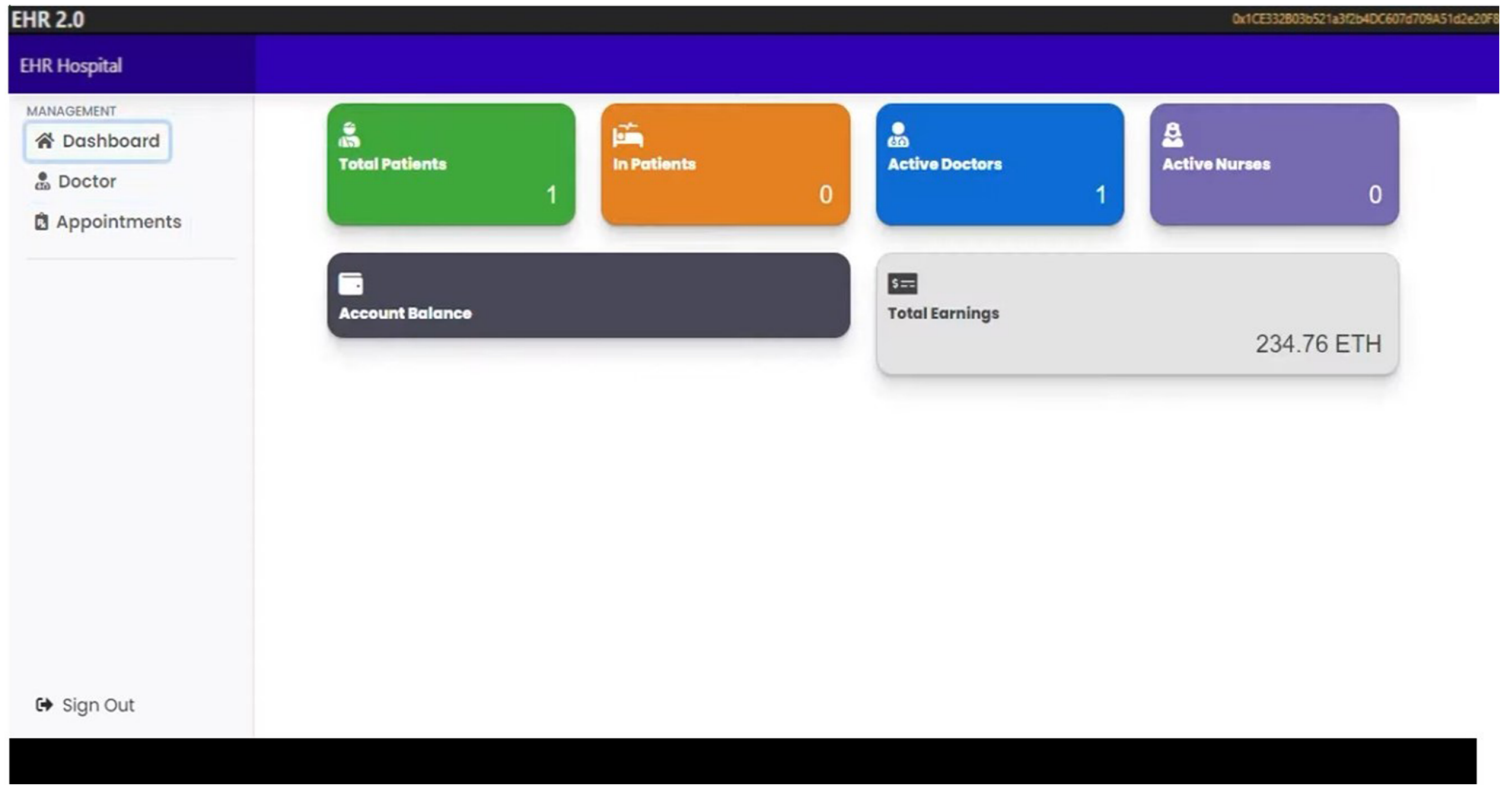
The admin dashboard.

Figure 5, the admin can add and view the doctor-patient, check appointments, and view the number of doctors and patients listed in the hospital.

**Figure 6:**
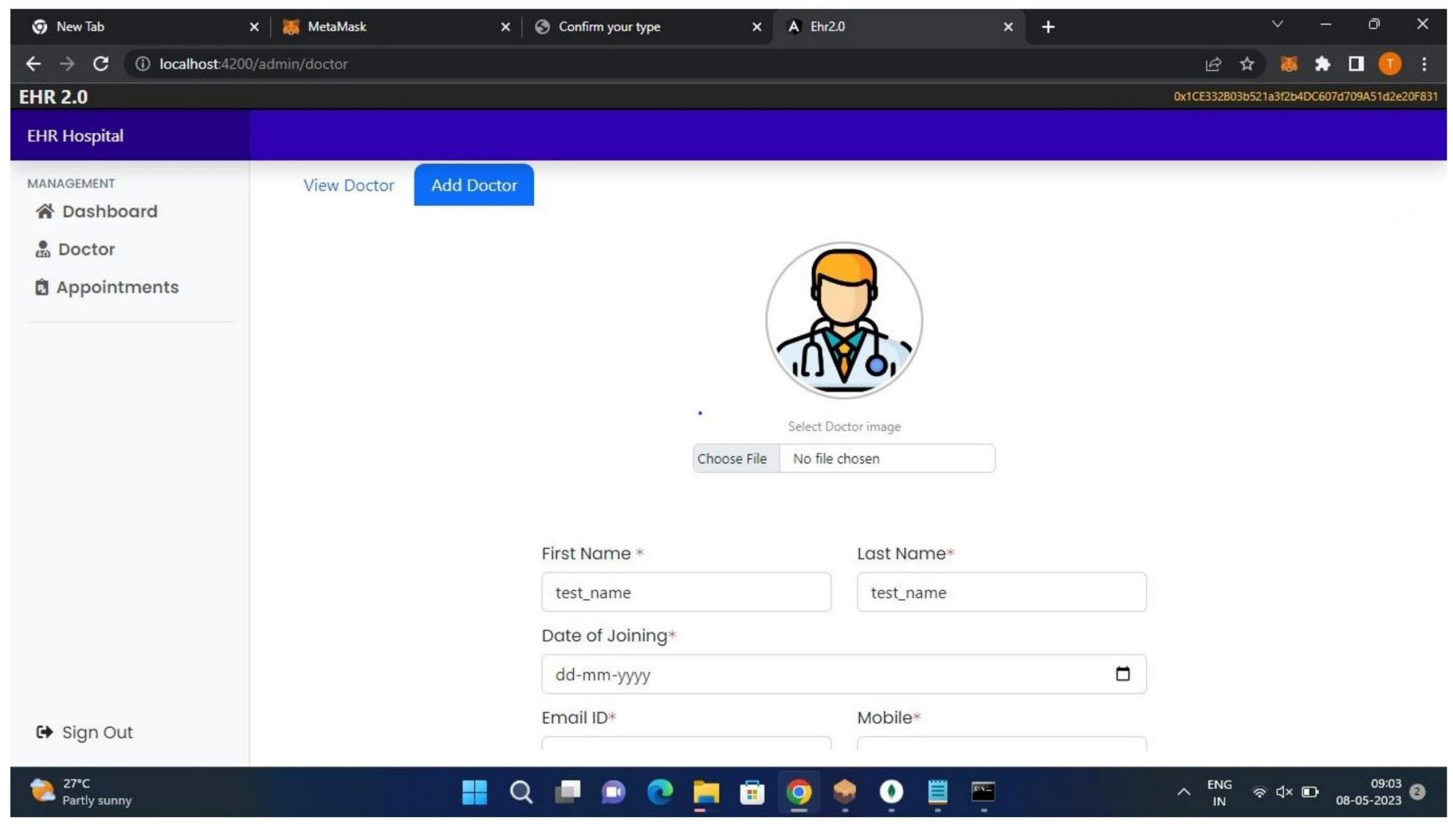
The admin module.

Figure 6, the admin can add data about doctors.

**Figure 7:**
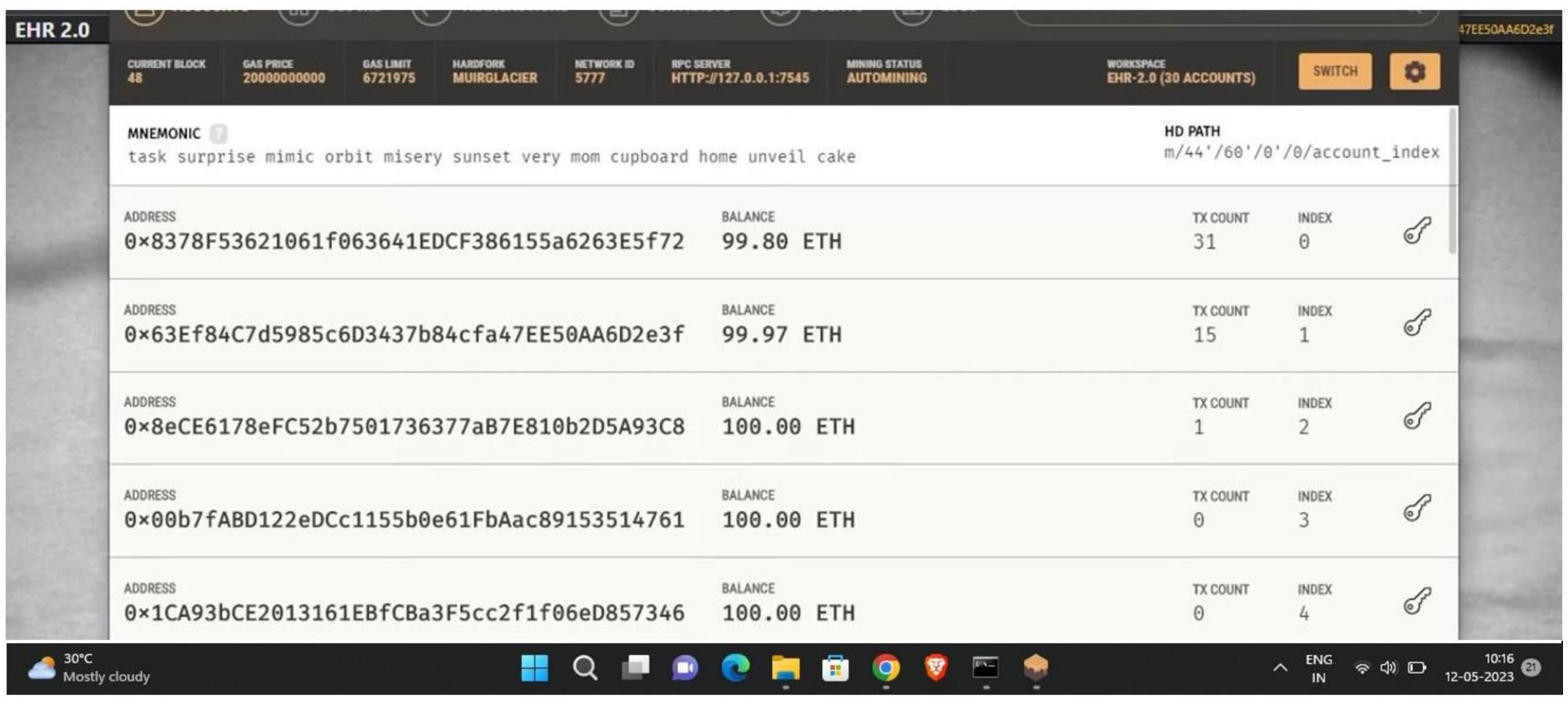
Ganache virtual ID.

Figure 7 shows all the generated EthereumVirtual IDs.

**Figure 8:**
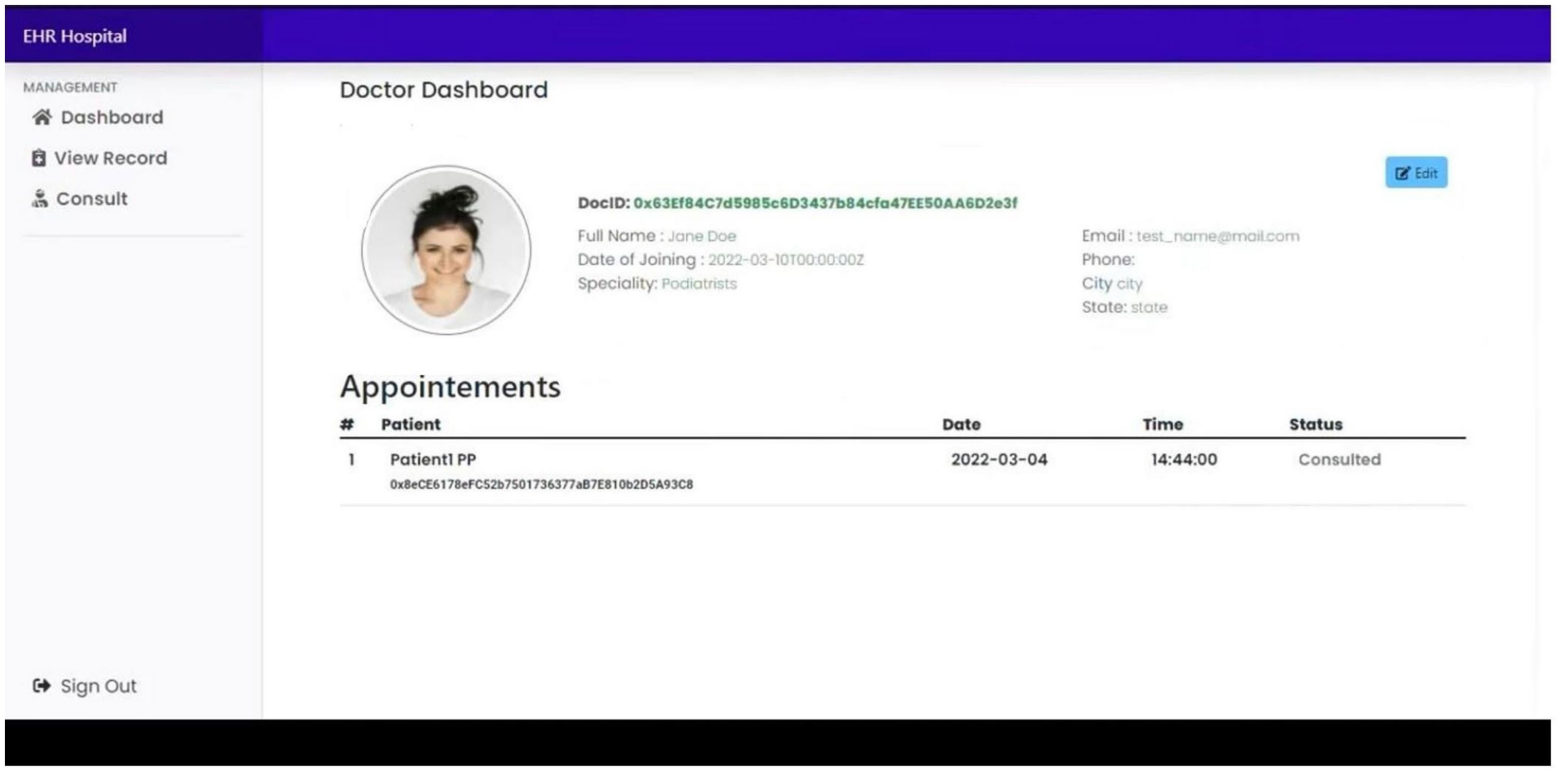
Doctor dashboard.

Figure 8, doctors can view patient records and their appointments.

**Figure 9:**
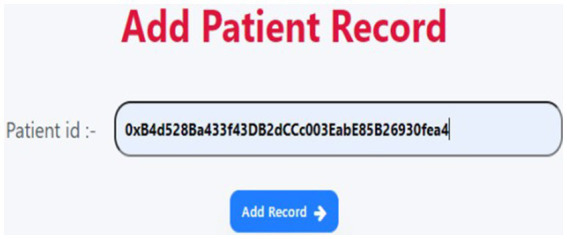
Add patient record.

Figure 9, the physician can observe the patient’s consultation and add a patient record by entering the patient’s account IDs.

**Figure 10:**
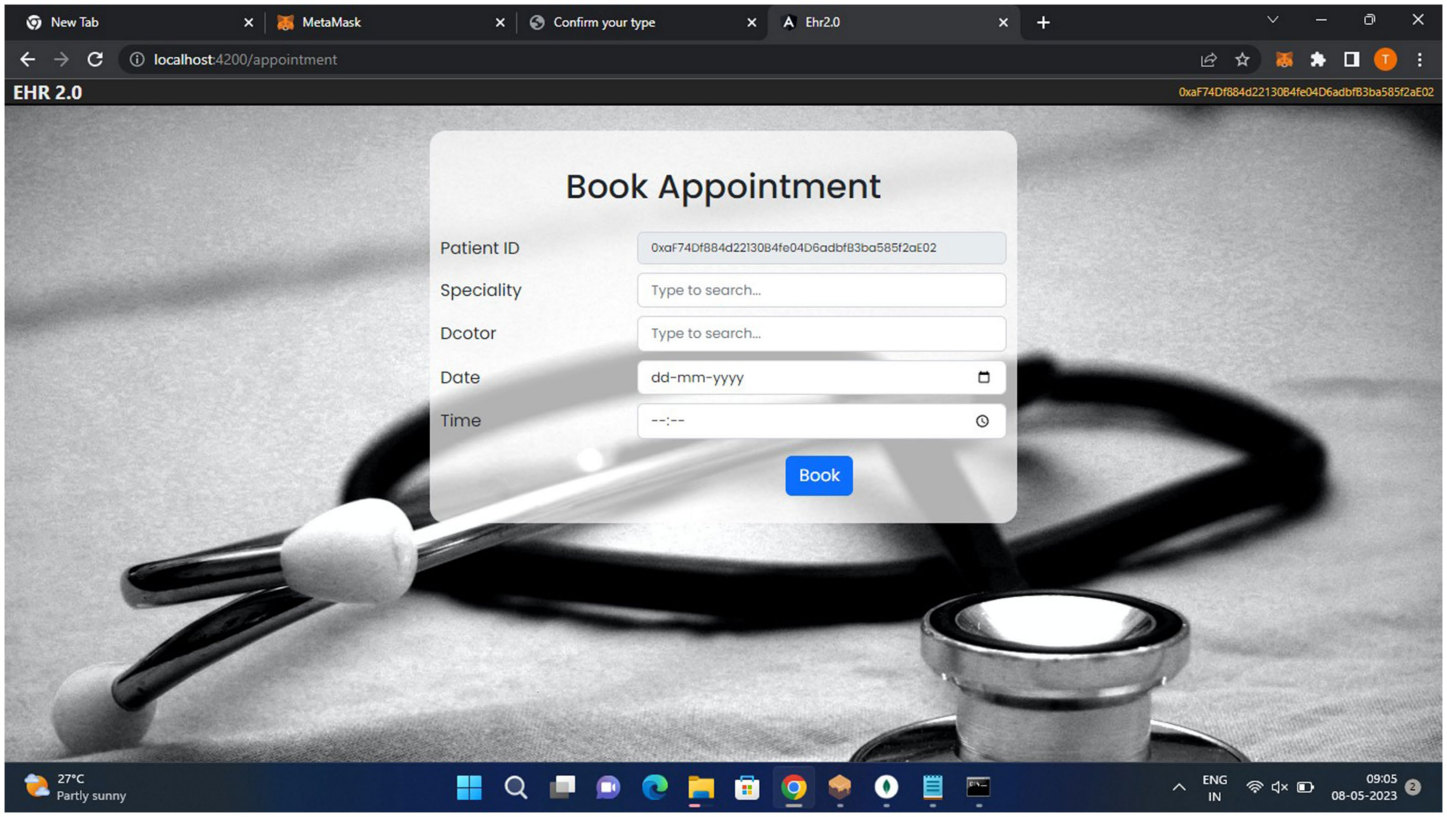
Patient appointment page.

Figure 10 helps the patient to book an appointment with their doctor.

**Figure 11:**
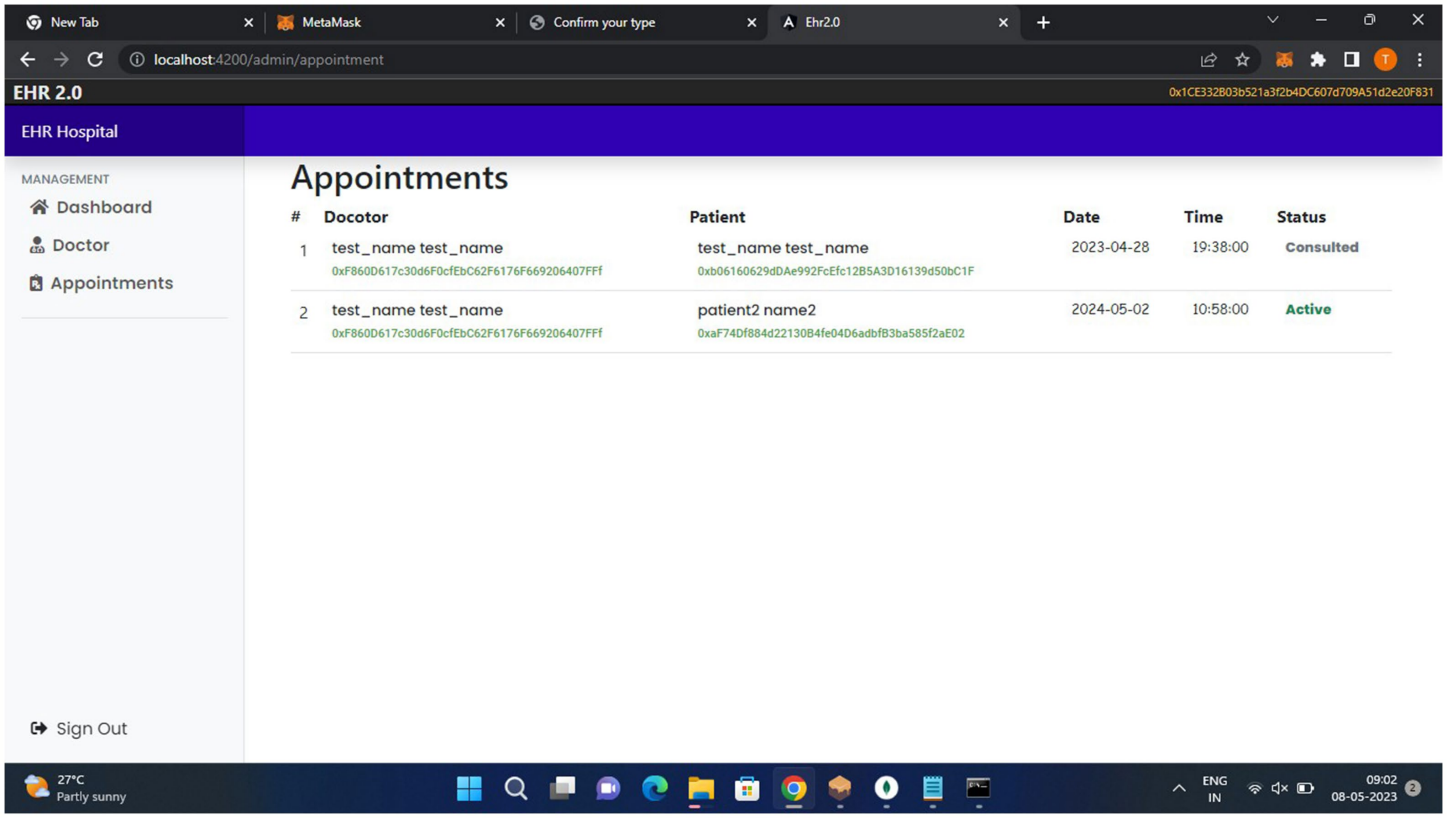
Patient module.

Figure 11, the patient can make their appointments and view their records.

### 5.4 User experience and adoption

We acknowledge the importance of user experience and adoption in the success of our EHR system. We have considered usability and user experience in our design and development process via, the following which is specified below.

#### 5.4.1 User-centered design

We employed user-centered design principles to create an intuitive interface for healthcare professionals and patients.

#### 5.4.2 Usability features

The proposed system includes usability features such as intuitive navigation and data visualization, streamlined workflows for efficient data entry and access, and customizable dashboards for personalized user experience.

#### 5.4.3 Adoption strategies

The proposed system includes adoption strategies such as training and support resources for healthcare professionals and user documentation.

### 5.5 Comparison of the framework proposal with related work

We already discussed some of the characteristics that our system employs and contrasted them with previous research in this area. Although these characteristics must be present in the framework, it is also believed that doing so will not jeopardize the system’s security and privacy.

#### 5.5.1 Scalability

Eberhardt et al. conducted a study to identify potential solutions for the blockchain’s scalability issue, and in this study, we argue that off-chain alternatives are necessary to enhance the functionality of current blockchain implementations and to lower usage costs while overcoming their constraints (31). Mohammed Misbhauddin suggested an architecture that lowers the cost of on-chain storage by utilizing an off-chain solution to lessen the high processing costs associated with big data blockchain transactions (32). Is it feasible to create an architecture or model based on blockchain technology that is both scalable and sufficiently secure for eHealth applications? Nabil and Rifi suggested architecture. They coupled the flexibility and significance of smart contracts with the scalability of off-chain databases and the security and privacy of the blockchain for upcoming eHealth DApps, all based on their expertise in blockchain technology and earlier works (33).

In simple words, scalability is the information system’s capacity to continue operating as intended as its storage capacity rises or falls. Scalability is a problem with blockchain technology that requires an ongoing solution when the volume or size of data on the blockchain grows. As the patient’s data stored on the blockchain includes the patient’s basic information in addition to the IPFS hash, that is, the off-chain scaling solution utilized in our suggested system framework, we employed the off-chain storage method in our proposed system. This resolves the scalability problem that has been raised since a significant number of patient medical records are currently not kept on the blockchain. Transactions could be completed more quickly as a result of the blockchain’s reduced data size. As was previously noted, IPFS uses cryptographic hashes that are stored over peer-to-peer networks in a decentralized fashion, guaranteeing that the framework’s security is maintained even when the scalability issue is resolved.

#### 5.5.2 Storage with addressable content

The IPFS off-chain storage method utilized in the suggested architecture is referred to as content-addressable storage. Since the patient’s sensitive record is kept on the IPFS, a hash of the record is generated. The patients and doctors can now access that hash when needed because it is now saved in the blockchain. The cryptographically secure hash that the IPFS creates guarantees the safety of the data that is kept on it. Furthermore, this guarantees the safety of our suggested structure.

#### 5.5.3 Role-based access control

This framework’s role-based access mechanism ensures that each entity in the system is assigned a role. The framework would remain inaccessible to any other party not granted authorization to use it. First, blockchain technology is secure in and of itself, and it employs specific protocols and mechanisms to keep itself safe from intrusions by external parties. This system offers two main forms of security. Furthermore, our system employs role-based access, which restricts access to the system and its features to individuals with defined roles. As a result, our solution would ensure that entities’ access to patient records is controlled in addition to their protection. This configuration further guarantees that the confidentiality of the patient’s private medical data is not compromised and that only permitted operators of the defined system has access.

#### 5.5.4 Evenness

The degree of trustworthiness and dependability of a system’s information storage are key indicators of its integrity. This technique, which is based on blockchain technology, ensures that this functionality is protected. Unauthorized parties have not altered the information kept by this system. Furthermore, only the individuals involved, including doctor, have access to the information.

#### 5.5.5 Confidentiality of information

Blockchain-based medical record storage should be shielded from outside access to preserve patient privacy. The patient’s data includes vital information about them, including their medical history, blood type, records, lab findings, X-ray reports, MRI results, and a host of other pertinent results and reports. This information is vital not only to the hospital but also to the patients. Smart contracts are a highly valuable component of this system because they guarantee accuracy, transparency, and confidence in the transactions that are being carried out. Only those who are trusted can access and view the records that are kept in the system. Any attempt to utilize the system by an untrusted third party is prohibited.

#### 5.5.6 Assessments of interoperability

Medical record interchange has been hampered by a major problem with interoperability in the healthcare sector. With the patient’s consent, an interoperability test was conducted in this study. The hospitals (Hospitals A, B, and C) were able to access and exchange the patient’s data by sending them a data request, and the patient could grant or revoke access to their medical records. The test of interoperability was accomplished. Moreover, users were able to share data error-free using a variety of browsers, including Edge, Firefox, Chrome, and Brave.

This framework would guarantee that the issue of privacy would be protected along with the confidentiality of the information against access by third parties. In comparison to other similar systems, we can infer that our suggested system offers higher and better efficiency and performance in terms of computing costs and communication compatibility. Furthermore, compared to previous systems, our suggested solution offers superior security features, such as scalability, RBAC, off-chain storage, decentralization, and interoperability. Table 4 compares the proposed framework with related work.

**Table 4:** Comparison of the proposed framework with related work.

Parameters in the proposed framework	Citations									Our proposed system
	Xia et al. (15)	Shahnaz et al. (17)	Margheri et al. (37)	Misbhauddin et al.(32)	Rifi et al. (33)	Atzei et al. (44)	Eltayieb et al. (45)	Wang et al. (46)	Guo et al. (47)	
Ethereum blockchain Based	Yes	Yes	Yes	Yes	Yes	Yes	No	No	No	Yes
Scalability	Yes	No	Yes	Yes	Yes	No	No	No	No	Yes
RBAC	No	No	No	No	No	yes	No	No	Yes	Yes
Off-chain storage	Yes	Yes	Yes	Yes	Yes	No	No	No	No	Yes
Decentralization	Yes	Yes	Yes	Yes	Yes	Yes	Yes	yes	Yes	Yes
Interoperability	No	No	No	No	No	No	No	No	No	Yes
Smart contract	Yes	Yes	Yes	Yes	Yes	Yes	No	No	Yes	Yes

## 6 Discussions and ethical considerations

### 6.1 Discussions

Here are some potential challenges and limitations of implementing the proposed EHR system in real-world settings:

- User adoption and training: Healthcare professionals’ willingness to adopt and effectively use the new system.
- Infrastructurer and resources: Adequate hardware, software, and network infrastructure to support the system.
- Regulatory compliance: Adhering to changing healthcare regulations, standards, and laws.
- Patient engagement and literacy: Ensuring patients understand and effectively use the system.
- Cost and funding: Significant investment in development, implementation, and maintenance.
- Change management: Managing cultural and organizational changes associated with adopting a new system.
- Data analytics and interpretation: Extracting meaningful insights from EHR data.
- System updates and maintenance: Regularly update and maintain the system to ensure continued functionality.

### 6.2 Ethical considerations

We recognize the sensitive nature of EHRs and the importance of protecting patient data privacy and obtaining informed consent.

#### 6.2.1 Data privacy

Our system employs robust security measures to ensure the confidentiality, integrity, and availability of patient data. Access controls and encryption techniques are used to protect patient data. We adhere to data minimization principles, collecting and processing only the necessary data for healthcare purposes.

#### 6.2.2 Informed consent

Patients provide explicit consent for data collection, storage, and sharing. Clear and transparent privacy notices explain how patient data is used and shared. Patients have the right to access, correct, or delete their personal health information.

#### 6.2.3 Compliance with regulations

Our system complies with relevant data protection regulations, such as HIPAA, GDPR, and CFR. We adhere to ethical guidelines for healthcare research and patient data usage.

#### 6.2.4 Ongoing monitoring and improvement

Regular security audits and risk assessments ensure the continued privacy and security of patient data. Patient feedback and concerns are addressed promptly, and our privacy and security practices are continuously improved. By prioritizing patient data privacy and informed consent, we uphold the trust placed in us as healthcare providers and protect the sensitive information entrusted to our care.

## 7 Conclusion and future enhancement

In this study, we propose a secure and interoperable EHR system leveraging blockchain and smart contracts. Our system ensures data integrity, confidentiality, and availability while enabling seamless sharing and collaboration among healthcare providers. We addressed key challenges in EHR management, including security, interoperability, and patient engagement. Our threat modeling and security analysis demonstrate the system’s robustness against potential threats.

Even though our proposed system significantly improves EHR management, there is still work to be done. For example, the system will need to be implemented in real-world healthcare settings to assess its efficacy and scalability. Usability studies will also need to be conducted to further improve the system’s user interface and experience. Advanced data analytics and artificial intelligence (AI) capabilities will also need to be developed to obtain insights from EHR data, and the system will need to be regularly assessed and improved to ensure it remains compliant with changing healthcare standards and regulations. Moreover, in the future, we plan to incorporate the payment module into the existing structure. The amount that a patient would pay for a consultation with the doctor on this decentralized blockchain system must be determined.

## Data Availability

The original contributions presented in the study are included in the article/supplementary material, further inquiries can be directed to the corresponding author/s.
